# COL8A1 Promotes NSCLC Progression Through IFIT1/IFIT3-Mediated EGFR Activation

**DOI:** 10.3389/fonc.2022.707525

**Published:** 2022-02-24

**Authors:** Xiangyi Zan, Shuyan Li, Shixiong Wei, Liping Gao, Lanting Zhao, Xiaoxia Yan, Yan Zhao, Junnian Shi, Yuping Wang, Rong Liu, Yuanyi Zhang, Yixin Wan, Yongning Zhou

**Affiliations:** ^1^ Department of Pneumology, The Second Hospital of Lanzhou University, Lanzhou, China; ^2^ Department of Gastroenterology, The First Hospital of Lanzhou University, Lanzhou, China; ^3^ Key Laboratory for Gastrointestinal Diseases of Gansu Province, Lanzhou University, Lanzhou, China; ^4^ Department of Chemistry and Chemical Engineering, Lanzhou University, Lanzhou, China; ^5^ Biological Science, University of California, Davis, Davis, CA, United States; ^6^ Clinical Medicine, University of South China, Hengyang, China

**Keywords:** NSCLC, COL8A1, IFIT1, IFIT3, EGFR

## Abstract

Activation of EGFR is a major risk factor for non-small cell lung cancer (NSCLC). Understanding the molecular events promoting EGFR activation can help us gain more insights into the progression of NSCLC. In this study, we demonstrate that collagen type VIII alpha 1 chain (COL8A1), an extracellular matrix component, was overexpressed in NSCLC. In NSCLC cells, knockdown of COL8A1 suppressed cell growth, cycle progression, and migration, and induced cell apoptosis. While COL8A1 overexpression promoted cell proliferation and inhibited cell apoptosis. In addition, we found that COL8A1 depletion reduced interferon response signaling and downregulated (IFIT1) and interferon-induced proteins with tetratricopeptide repeats 3 (IFIT3). Moreover, we indicated that COL8A1 could upregulate IFIT1 and IFIT3 mediated EGFR activation *in vitro* and *in vivo.* Lastly, there was a positive correlation among COL8A1, IFIT1, and IFIT3 expression, and EGFR activity in patients with NSCLC. Overall, our data demonstrate that COL8A1 contributes to NSCLC proliferation and invasion through EGFR activation, dependent on IFIT1 and IFIT3 expression.

## Introduction

Non-small cell lung cancer (NSCLC) is one of the most frequent malignancies, with high incidence and mortality rates worldwide (1). Many patients with NSCLC present with metastatic disease at diagnosis and have poor prognosis (2). Resistance to antitumor therapies is the main cause of the gradual increase in mortality due to NSCLC (3). Numerous genetic elements are involved in the pathogenesis and progression of NSCLC, including EGFR and K-Ras (4–6), which function as oncogenes and play important roles in human cancer development. Further, dysregulation and mutations within Collagen type VIII are common mechanisms driving tumorigenesis (7); however, understanding of the roles of Collagen type VIII in NSCLC is limited, highlighting an urgent need for further investigation.

COL8A1 was initially identified as a major class of collagen type VIII, expressed by cornea and vascular endothelial cells, in the 1980s (8) and was then further characterized as a tumor suppressor in hepatocarcinoma cells (9). It is now evident that COL8A1 has important roles in various biological processes, and its dysregulation has been linked to various cancers, including hepatic carcinoma and hemangioma (10). Increasing evidence indicates that COL8A1 functions as a pivotal player in atherogenesis and vascular remodeling, partly *via* controlling cell proliferation and invasion (11). Additionally, COL8A1 serves as novel non-fibrillar collagen that is overexpressed in dilated cardiomyopathy, which is associated with left ventricular mass index and involved in the progression of cardiac dilation and remodeling (12). One report suggested that COL8A1 was significantly upregulated and could be a useful marker for early diagnosis of adamantinomatous craniopharyngioma (13). Further, COL8A1 expression can be used to segregate gastrointestinal stromal tumors into different tumor behavior groups (14). Although the function of COL8A1 is not completely elucidated, its dysregulation in various cancers has led to a proposed role in NSCLC development and progression.

Interferons (IFNs) were initially described as agents interfering with viral multiplication and exerting potent anti-viral activity, and are now understood as pleiotropic cytokines that promote hundreds of interferon-stimulated genes (ISGs) (15). The IFN-γ signaling pathway and ISGs are associated with tumor occurrence and development, as well as invasion (16–18). Among ISGs, human interferon-induced proteins with tetratricopeptide repeats genes (IFITs), including *IFIT1*, *IFIT2*, and *IFIT3* encode major factors with anti-virus activities (19, 20). Recently, cumulative evidence has revealed that IFITs are involved in tumor progression (21). IFIT1 and IFIT3 contribute to the metastasis of oral squamous cell carcinoma, and serve as prognostic markers for patients with this disease. Nevertheless, the molecular functions of IFIT1 and IFIT3 in NSCLC development have not been fully explored.

In the present study, we firstly determined whether COL8A1 has important roles in NSCLC development. Results based on analysis of clinical NSCLC and paired adjacent tissue samples clearly showed that COL8A1 was upregulated in NSCLC, and contributed to cell proliferation, invasion, and cycle progression, as well as suppressing apoptosis of NSCLC cell lines. Further, to explore the mechanisms of COL8A1 promoted lung cancer development, we found COL8A1 activated EGFR *via* upregulation of IFIT1 and IFIT3. Overall, COL8A1 upregulation of IFIT1 and IFIT3 contributes to NSCLC development through activation of EGFR.

## Materials and Methods

### Patients and Specimens

A total of 94 clinical NSCLC samples and 86 adjacent tissues were enrolled in this study at the Second Hospital of Lanzhou University between 2017 and 2020. NSCLC samples were diagnosed by two independent pathologists. This investigation was performed according to the World Medical Association Declaration of Helsinki and approved by the Ethics Committee of the Second Hospital of Lanzhou University.

### Cell Lines, Lentiviral-Mediated COL8A1 Knockdown and COL8A1 Overexpression

A549, H1299 and H1975 NSCLC cell lines were purchased from ATCC and then cultured in RPMI 1640 (Hyclone, USA), supplemented with 10% fetal bovine serum (Gibco, USA), antibiotics (100 U/mL, 1× penicillin/streptomycin, Gibco), and routinely maintained at 37°C in 5% CO_2_. The GV115 (pGCSIL-GFP; Shanghai GeneChem Co., Ltd.) lentivirus was used for constructing COL8A1-knockdown vectors. Targeted sequences were as follows: shCOL8A1-1, 5’-GCCUAUGAGAUGCCUGCAUUUdTdT-3’; shCOL8A1-2, 5’-GCCACCACAAAUUCCACAAUAdTdT-3’; shCtrl, 5’-TTCTCCGAACGTGTCACGT-3’. The shCOL8A1 or shCtrl expression vectors, combined with Helper 1.0 and Helper 2.0 packaging vectors, were co-transfected into 293T cells using Lipofectamine™ 2000. The virus supernatants were collected by ultracentrifugation. After 72 hours transfection, the cells was used for subsequent experiments. For overexpression, the coding sequence of COL8A1 (NM_001850.5), were cloned into pCDNA3.1 vectors. The plasmids were transfected in to cells using Lipofectamine 3000 (Invitrogen). After 48 hours transfection, the cells were used for subsequent experiments.

### RNAi Interference

siRNA was used to interfere iFIT1, iFIT3 or COL8A1 in A549, H1299 and H1975 cells. siRNAs against negative control, iFIT1 and iFIT3 were purchased from Huzhou Hippo Biotechnology Co., Ltd. and were transfected into A549, H1299 and H1975 cells by RNAiMAX (Invitrogen). 48 hours later, the cells were subjected to immunoblotting analysis of knockdown efficiency and functional experiments. Targeted sequences were as follows: siIFIT1-1, 5’-CUUCGGAGAAAGGCAUUAGAUdTdT-3’; siIFIT1-2, 5’-CGUCAAUGCAAUUAUCCAUUAdTdT-3’; siIFIT3-1, 5’-GCAAUAUGCUAUGGACUAUdTdT-3’; siIFIT3-2, 5’-GCAAGCAGCCAAAUGUUAUdTdT-3’; siCOL8A1-1, 5’- GCCUAUGAGAUGCCUGCAUUUdTdT-3’; siCOL8A1-2, 5’- GCCACCACAAAUUCCACAAUAAdTdT-3’; siCtrl, 5’-UUCUCCGAACGUGUCACGUdTdT-3’.

### Immunohistochemistry

To quantify the expression of selected proteins, formalin-fixed paraffin-embedded tissue sections were analyzed by immunohistochemistry (IHC). Formalin-fixed, paraffin-embedded (FFPE) tissue samples were deparaffinized and rehydrated, then followed by heat-induced antigen retrieval. And then stained using primary anti-COL8A1 antibody (bs-7529R, Bioss antibodies), anti-iFIT1 (23247-1-AP, Proteintech), anti-iFIT3 (15201-1-AP, Proteintech) or anti-pEGFR (#3777S, CST). The intensity of expression was graded as follows: negative = score 0, weak = score 1, moderated = score 2 and strong = score 3. Low expression means refers to a score 0 or 1, and high expression means refers to a scoring score 2 or 3. Immuno-reactivity was assessed independently by two expert pathologists blind to all clinical data.

### Cell Viability Assay

A Celigo Image Cytometer (Nexcelom) or CCK8 assay were used to determine the proliferation capacity of NSCLC cell lines, according to the manufacturer’s instructions. Celigo Image Cytometer (Nexcelom) was used in COL8A1 knockdown experiment. Briefly, H1299, A549 and H1975 cell lines (2.5 × 10^3^ cells/well) were seeded into 96 well plates, with three replicates of each treatment group, and then maintained at 37°C for 5 days. The number of cells with green fluorescence was calculated by Celigo cell count every 24 h after cell seeding.

CCK8 assay was used in the rest of cell viability assay. Briefly, a total of 1000 cells were seeded into 96-well plates. At indicated time, 10 μl of CCK8 regent was added into each well followed by incubation at 37°C for 3 hours. Then OD value at 450 nm was measured.

### Colony Formation Assay

NSCLC cells (5 × 10^3^ cells/well/100 µl) subjected to corresponding treatments were seeded into 6-well plates and incubated for 10 days. At the end of experiments, cells were washed with PBS three times, fixed in 4% paraformaldehyde for 10 min, then stained with 1% crystal violet. After air-drying, the numbers of colonies > 2 mm diameter were quantified.

### Transwell Migration & Invasion Assays

For the transwell assay (Corning Costar, USA), H1299, A549 and H1975 cells (density, 1.5 × 10^5^) were seeded on the noncoated membrane (for cell migration assay) or the Matrigel-coated membrane (for cell invasion assay) of upper chambers of 24-well units without serum, and 0.6 mL of medium supplemented with 10% FBS added to the lower chambers to serve as a chemoattractant. After culture for 18 h, cells that had not migrated to the lower surface of the chamber were completely removed using a cotton swab. Then, the chambers were fixed with methanol for 15 min and then stained with crystal violet at room temperature. The invasive cells were counted under a light microscope, with at least five random fields analyzed for each sample. The experiments were repeated three times.

### Apoptosis Assay

Apoptosis was examined by flow cytometry. Briefly, cells subjected to different treatments were harvested, washed twice with cold PBS, and then incubated with Annexin V-FITC, or Annexin V-APC and propidium iodide (PI) staining solution (Shanghai YEASEN Biotech Co. Ltd) for 30 min at 37°C in dark conditions, following the manufacturer’s instructions. Annexin V-APC and propidium iodide (PI) staining was used in COL8A1 knockdown experiment, while Annexin V-FITC and propidium iodide (PI) staining was used in the rest of apoptosis assay. To induce cell apoptosis, the cells were treated with 2uM PAC-1 (Selleck, S2738). Then the cells were subjected to cell apoptosis assay. Cells were analyzed by flow cytometry (cytoFlex, Beckman Coulter, Inc.). The data were analyzed by Cytexpert (Version 2.4.0.28, Beckman Coulter, Inc.).

### Cell Cycle Assay

Cells were infected with the shCOL8A1 or shCtrl lentivirus. Cells were fixed with 70% cold ethanol at 4°C overnight, washed twice with ice-cold PBS, and incubated with 10 mg/ml RNase at 37°C. The cell cycle was monitored by propidium iodide (PI) staining of the nucleus. The fluorescence of DNA-bound PI in cells was measured by flow cytometry (FACS Calibur, Becton Dickinson).

### qRT-PCR

In brief, total RNA was extracted using Trizol regent (Invitrogen) and subsequently digested with DNase enzyme (Invitrogen). qRT-PCR assays were conducted using a SYBR master mixture Kit (TaKaRa) on a StepOne Real-Time PCR system (Thermo Fisher). Primer sequences are presented in **Supplementary Table 1**. Relative RNA expression levels were normalized to those of β-actin. The Ct2-ΔΔCt method was performed to examine the relative expression.

### Western Blotting

Whole cell lysates were extracted using RIPA reagent (Beyotime, China) supplemented with protease inhibitors (Roche cOmplete ULTRA tablets), and protein concentrations determined using a Bicinchoninic Acid Protein assay Kit (Thermo Fisher, USA). Total protein aliquots (20 μg) were separated by SDS-PAGE, as described previously. Primary antibodies against p-EGFR (#3777S, CST), EGFR (#2085, CST), p-AKT (#4060, CST), AKT (#4821, CST), IFIT-1 (23247-1-AP, Proteintech), IFIT-3 (15201-1-AP, Proteintech), and β-actin (#4970, CST) were applied in this study. Secondary antibodies used were IRDye 800CW Goat anti-rabbit 926-32211 (Lot No. C90723-19, LI-COR) and IRDye 800CW Goat anti-mouse 926-32210 (Lot No. C90408-08, LI-COR).

### Tumor Growth *In Vivo*

To evaluate the effects of COL8A1 expression on the growth of NSCLC cells *in vivo*, a nude mouse xenograft model was used. Female BALB/c nude mice (6–8 weeks old) were raised in a specific pathogen-free environment. Stable A549 cell lines expressing shCOL8A1 or control shRNA were sorted and maintained in growth medium supplemented with puromycin (2 µg/mL) for one week. NSCLC A549 cells transfected with shCtrl or shCOL8A1 (2 × 10^6^ cells/mouse) were injected subcutaneously into the right flanks of mice. After inoculation, tumor volume was evaluated by caliper measurements every 3 days and calculated according to the formula: volume = 1/2 × length × width^2^. When tumor volumes in the shCtrl group reached 600 cm^3^, mice were killed and tumors harvested for analysis.

### Statistical Analysis

Results are presented as the mean ± standard deviation. Statistical differences between two groups were determined using a Student’s t-test. Statistical differences among ≥3 groups were determined using a one-way ANOVA followed by a Tukey’s *post hoc* test. Categorical data were analyzed using a Chi-Square test. Spearman’s correlation coefficients (rs) were calculated between COL8A1 staining score and IFIT1, IFIT3 or p-EGFR staining score. P < 0.05 was considered statistically significant.

## Results

### COL8A1 Is Upregulated in NSCLC

**Figure 1:**
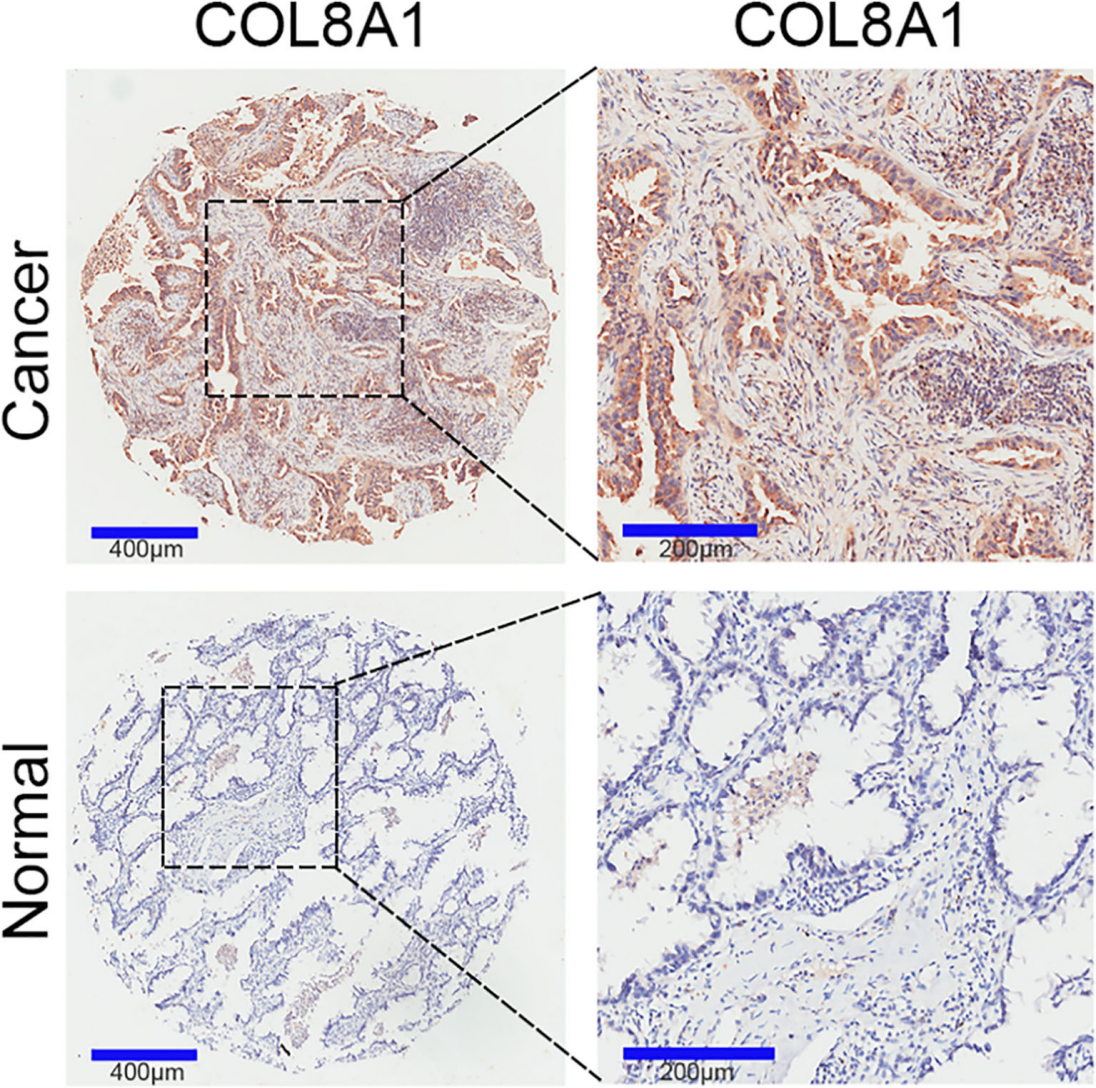
COL8A1 overexpression in NSCLC tissues. COL8A1 expression in 94 NSCLC and 86 paired adjacent normal tissue samples was determined by IHC assay.

To identify and characterize COL8A1 in NSCLC, we examined its expression levels in NSCLC and paired adjacent normal tissue samples. The results revealed that COL8A1 was highly expressed in NSCLC tissues. Moreover, of the 94 cases included in the IHC staining assay, COL8A1 was positively expressed in 74 NSCLC tissues (78.7%) (**Figure 1** and **Table 1**). To assess possible associations between COL8A1 expression and tumor characteristics, IHC scores for COL8A1 were analyzed in relation to clinicopathological parameters. As shown in **Table 2**, high expression of COL8A1 was strongly associated with more advanced pathologic stage of NSCLC (II–III), compared to earlier pathologic stage (I). These results suggest that COL8A1 may be acted as an important factor in the development of NSCLC.

**Table 1:** The expression of COL8A1 in Normal tissue and NSCLC by IHC.

	Tumor Tissue	Adjacent tissue	χ^2^	p Value
COL8A1 high expression	69	10	69.60	<0.001
COL8A1 low expression	25	76		
Total	94	86		

**Table 2:** Correlation of COL8A1 expression with clinicopathological characteristics in 94 patients of NSCLC.

	Characteristic	COL8A1 expression		p Value
		High	Low	
Age	<60	32	12	0.889
	≥60	37	13
				
Gender	Male	37	16	0.37
	Female	32	9
				
Stage I/II/III	I	5	6	0.026
	II/III	64	19
				
T classification	T0-T1	51	20	0.544
	T2-T3	18	2
				
Number of lymph nodes	≤9	42	10	0.072
	>9	27	15
				
Tumor size	<60	54	15	0.077
	≥60	15	10

### COL8A1 Stimulates NSCLC Cell Proliferation and Migration

**Figure 2:**
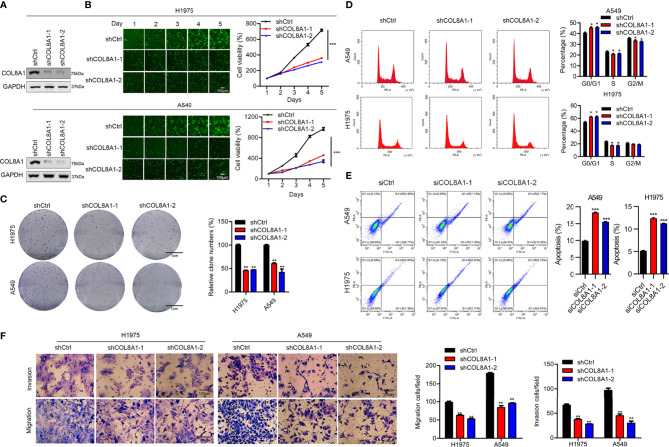
Knockdown of COL8A1 suppresses NSCLC cell line progression. **(A)** Knockdown efficiency of lentivirus-mediated COL8A1-knockdown in A549 and H1975 cells determined by western blot assay. **(B)** Cell proliferation curves generated using cell viability assays. **(C)** Representative image of the results of colony formation assays, showing crystal violet staining. **(D)** Cell cycle analysis examined by flow cytometry. **(E)** Cell apoptosis assays of A549 and H1975 NSCLC. **(F)** Cell migration and invasion capacity of COL8A1-silenced NSCLC cells determined by transwell assays of A549 and H1975 cells. *p < 0.05. **p < 0.01. ***p < 0.001.

Our clinicopathological data clearly indicated that COL8A1 was associated with tumor pathological stage. To determine the role of COL8A1 in NSCLC, the cell lines, A549 and H1975, were transfected with lentivirus encoding COL8A1 shRNA (shCOL8A1-1 and shCOL8A1-2) and empty lentiviral vectors (shCtrl) to establish stable COL8A1-silenced cell lines. As shown in **Figure 2A**, COL8A1 was clearly suppressed in both A549 and H1975 cell lines. Knockdown of COL8A1 markedly suppressed the capacities of proliferation and colony formation in A549 and H1975 cells (**Figures 2B, C** and **Supplementary Figures 1A–C**). Cell cycle analysis indicated that A549, H1975 and H1299 cells expressing shCOL8A1 induced cell cycle arrest at G1 phase (**Figure 2D** and **Supplementary Figure 1D**). Further, we found that COL8A1 knockdown promoted apoptosis in A549, H1299 and H1975 NSCLC cell lines, indicating that silencing of COL8A1 could partly suppress cell growth by promoting apoptosis (**Figure 2E** and **Supplementary Figure 1E**).

As tumor progression and metastasis is a complex process involving cell motility, including cell invasion and migration, we next examined the effects of COL8A1 expression on NSCLC cell invasion using transwell assays. COL8A1-silenced A549 and H1975 cells exhibited significantly lower invasion ability compared with shCtrl cells (**Figure 2F**, **Supplementary Figure 1E**).

**Figure 3:**
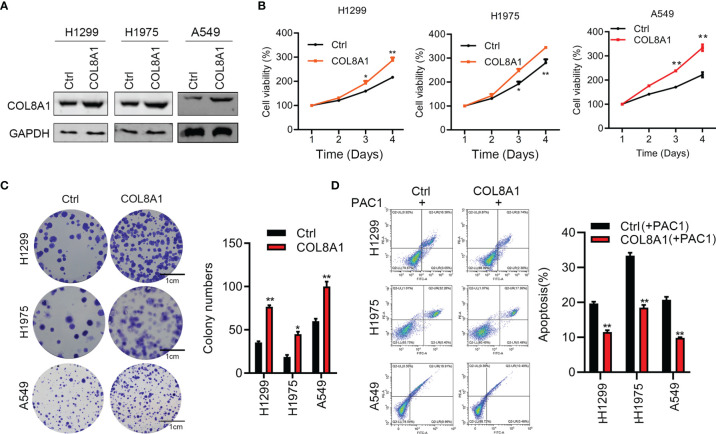
Overexpression of COL8A1 contributes to cell proliferation, invasion and cell cycle of NSCLC cells. **(A)** The efficacy of COL8A1 overexpression was examined by Western blot. **(B)** Effects of COL8A1 overexpression on cell proliferation were examined by cell viability assay in A549, H1299 and H1975 cell lines. **(C)** Effects of COL8A1 overexpression on colony formation in A549, H1299 and H1975 cell lines. **(D)** Flow cytometry was performed to examine cell apoptosis of COL8A1-overexpressing NSCLC cells. *p<0.05. **p<0.01.

To further explore its roles acted as an oncogene, COL8A1 was overexpressed in A549, H1299 and H1975 cells, as confirmed by western blotting (**Figure 3A**). As shown in **Figures 3B, C**, COL8A1-overexpressing cells displayed markedly enhanced cell proliferation and colony formation capacities relative to control cells. To explore the apoptosis activity medicated by COL8A1, lung cancer cells were treated with an apoptosis inducer, PAC-1, and the results indicated that COL8A1 overexpression significantly suppressed apoptosis of A549, H1299 and H1975 cells (**Figure 3D**). These results demonstrate that COL8A1 overexpression promotes cell proliferation and colony formation, as well as suppressing apoptosis, of A549, H1299 and H1975 cells.

### COL8A1 Upregulates IFIT1 and IFIT3 and Activates EGFR in NSCLC Cells

**Figure 4:**
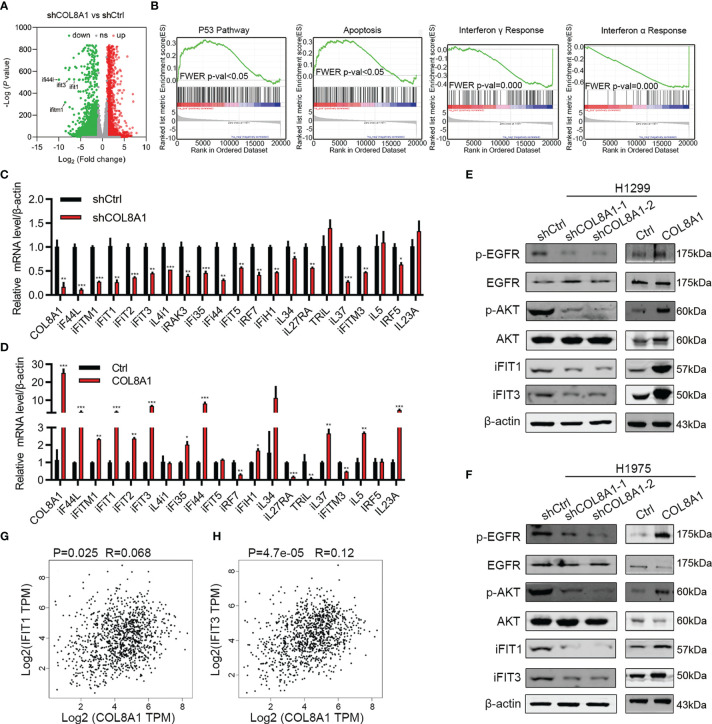
COL8A1 stimulates IFIT1 and IFIT3 expression and EGFR activity in NSCLC cells. **(A)** RNA-seq data showing dysregulated genes in COL8A1-silenced NSCLC cells compared with shCtrl cells. **(B)** Gene set enrichment analysis (GSEA) of genes regulated in COL8A1-silenced and control cells. **(C, D)** qRT-PCR analysis of dysregulated genes in COL8A1-silenced and COL8A1-overexpressing cells. **(E, F)** Western blot evaluation of the effects of COL8A1-silencing and COL8A1-overexpression on the IFIT pathway and EGFR activity in NSCLC cells. **(G, H)** Correlation analysis between COL8A1 and IFIT1 or IFIT3 expression. *p < 0.05. **p < 0.01. ***p < 0.001.

To further explore the mechanisms underlying COL8A1 involvement in NSCLC progression, we applied RNA-seq technology to determine whether COL8A1 knockdown influenced gene expression in NSCLC cells. RNA-seq results identified 3959 up-regulated and 5542 down-regulated genes in COL8A1 knockdown cells (**Figure 4A**), which were involved in apoptosis, p53, interferon alpha response signaling and interferon gamma responses, as well as the interferon alpha response pathway (**Figure 4B**). To confirm this finding, we detected the related gene expression in interferon alpha response signaling and interferon gamma response signaling by qPCR, the results demonstrated that the expression of genes related to interferon signaling were significantly changed after COL8A1 knockdown or COL8A1 overexpression (**Figures 4C, D**).

Activation of interferon signaling has vital roles in regulating NSCLC progression. In addition, IFIT1 and IFIT3 are critical for EGFR recycling and activation in other cancers (21). Hence, whether COL8A1 upregulation of IFIT1 and IFIT3, activates EGFR in NSCLC warrants investigation. Notably, COL8A1 knockdown in H1975 and H1299 cells robustly suppressed the expression of IFIT1, IFIT3, and phosphorylation of AKT and EGFR (**Figures 4E, F**). Conversely, COL8A1 overexpression remarkably increased the levels of phosphorylated of AKT and EGFR, as well as of IFIT1 and IFIT3, in H1975 and H1299 cells (**Figures 4E, F**). Moreover, according to correlation analysis using data from NSCLC tissues in TCGA database, *IFIT1* and *IFIT3* expression levels were positively correlated with those of COL8A1 (**Figures 4G, H**). Taken together, these results suggested that COL8A1 may activate EGFR by upregulating IFIT1 and IFIT3.

### IFIT1 or IFIT3 Knockdown Can Rescue the Effects of COL8A1 on Cell Proliferation, Migration, and Apoptosis in NSCLC Cell Lines

**Figure 5:**
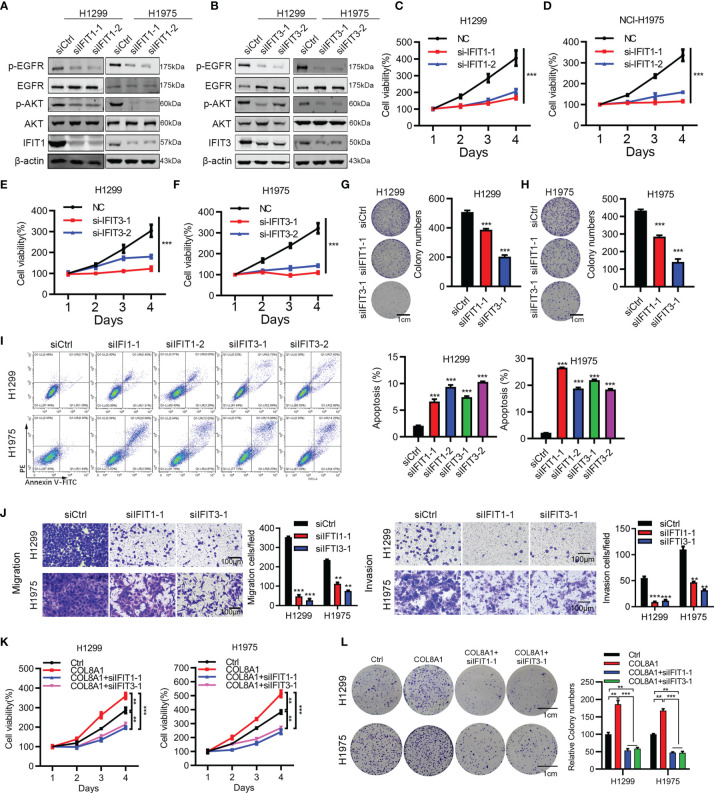
IFIT1 and IFIT3 knockdown effectively suppresses proliferation, migration, and apoptosis of NSCLC cells. **(A, B)** Effects of IFIT1 and IFIT3 knockdown on EGFR and AKT activity determined by western blotting. **(C, D)** Cell viability assays performed in IFIT1-knockdown H1299 and H1975 cells. **(E, F)** Cell viability assays performed in IFIT3-knockdown H1299 and H1975 cells. **(G, H)** Colony formation assessed in siCtrl-, siIFIT1-1, and siIFIT3-1-treated H1299 **(G)** and H1975 **(H)** cells. **(I)** Effects of IFIT1 and IFIT3 knockdown on apoptosis assessed by flow cytometry. **(J)** Effects of IFIT1 and IFIT3 knockdown on cell invasion and migration assessed by transwell assay. **(K, L)** H1299 and H1975 cells transfected with COL8A1, siIFIT1, and siIFIT3 were subjected to cell viability **(K)** and colony formation analysis **(L)**. **p < 0.01. ***p < 0.001.

Next, we aimed to investigate the functional roles of IFIT1 and IFIT3 in NSCLC development. Western blotting showed that silencing of IFIT1 and IFIT3 effectively suppressed their expression and inhibited AKT and EGFR activity (**Figures 5A, B**). In addition, to explore the mechanism of COL8A1 upregulated IFIT1 and IFIT3-medicated EGFR activation, we overexpressed COL8A1 in lung cancer cells H1299 and H1975, and found that IFIT1, IFIT3 and pEGFR were upregulated by COL8A1 overexpression, whereas after co-transfected with COL8A1-overexpressing vectors and siIFIT1/siIFIT3, the expression of IFIT1, IFIT3 and pEGFR were suppressed, which indicated that COL8A1 promoted EGFR activation through upregulating IFIT1 and IFIT3 in lung cancer cells (**Supplementary Figure 2**). Cell viability and colony formation assays suggested that knockdown of IFIT1 and IFIT3 significantly suppressed proliferation of H1299 and H1975 cells (**Figures 5C–H**). In addition, silencing of IFIT1 and IFIT3 inhibited NSCLC cell migration and invasion, and enhanced NSCLC cell apoptosis (**Figures 5I, J**).

To further investigate whether COL8A1-mediated tumor progression required IFIT1 and IFIT3 knockdown, we examined cell proliferation in COL8A1-overexpressing and IFIT1/IFIT3-silenced NSCLC cells. As shown in **Figures 5K, L**, IFIT1 or IFIT3 knockdown efficiently blocked COL8A1-enhanced NSCLC cell proliferation and colony formation capacity. Collectively, either IFIT1 or IFIT3 could restore the effects of COL8A1 on cell proliferation and migration in H1299 and H1975 cells.

### COL8A1 Contributes to NSCLC Progression *In Vivo*

**Figure 6:**
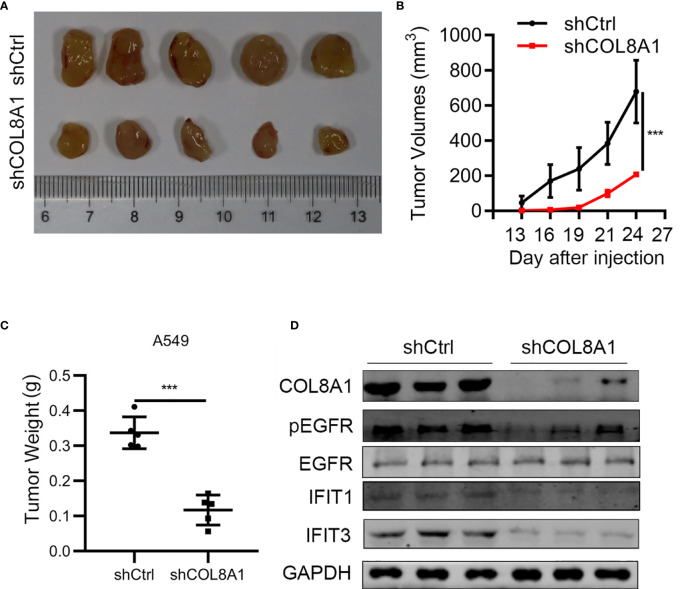
COL8A1 contributes to NSCLC progression by activating EGFR *in vivo*. BALB/c nude mice were subcutaneously transplanted with COL8A1-silenced NSCLC cells. Mice were sacrificed 5 weeks after implantation. **(A)** Macroscopic images of xenografted tumors. **(B)** Tumor growth curves. **(C)** Tumor weight was determined immediately after sacrificing the mice. **(D)** Tumor tissues were subjected to western blotting analysis with the indicated antibodies. ***p < 0.001.

To further explore the function of COL8A1 *in vivo*, we established a mouse xenograft model by subcutaneously injecting shCOL8A1-expressing cells into nude mice. COL8A1-silenced xenograft tumors grew significantly more slowly than those in the control group, with tumor volume and weight in COL8A1-silenced tumors lower than those expressing control shRNA, suggesting that knockdown of COL8A1 suppressed tumor growth capacity *in vivo* (**Figures 6A–C**). Western blot analysis of tumor tissues showed that COL8A1 knockdown reduced IFIT1 and IFIT3 expression, as well as EGFR phosphorylation (**Figure 6D**). Overall, our data demonstrate that COL8A1 is critical for NSCLC growth *in vivo*.

### COL8A1 Expression Is Positively Correlated With That of IFIT1/IFIT3 and EGFR Phosphorylation

**Figure 7:**
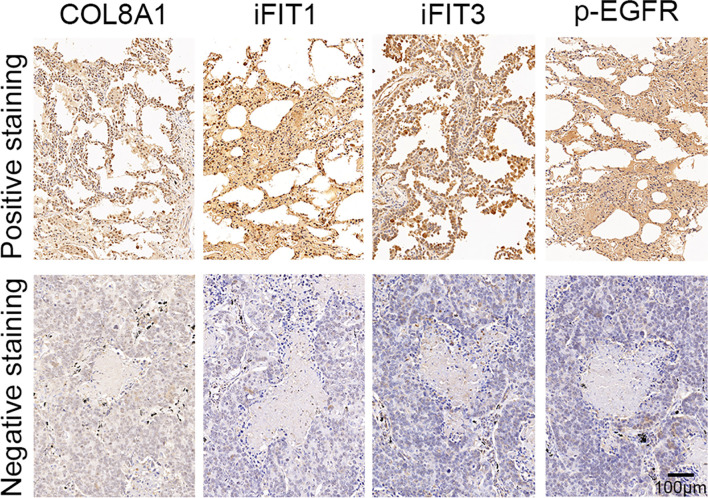
Analysis of COL8A1, IFIT1, IFIT3, and p-EGFR in NSCLC tissues. The expression of COL8A1, IFIT1, IFIT3, and the phosphorylation of EGFR in NSCLC tissues detected by IHC staining.

Finally, we assessed the correlations between COL8A1 and IFIT1/IFIT3 expression levels, and EGFR phosphorylation. To this end, a total of 50 NSCLC tissue samples were subjected to IHC staining analysis, which demonstrated that COL8A1 protein abundance was positively correlated with IFIT1/IFIT3 expression and EGFR phosphorylation (**Figure 7** and **Table 3**). A positive Spearman correlation co-efficient was also detected between IFIT1/3 expression and EGFR activity in human NSCLC samples (**Figure 7** and **Table 3**). Therefore, COL8A1 activation of EGFR through upregulation of IFIT1/IFIT3 may occur in human NSCLC.

**Table 3:** Spearman correlation analysis of expression among COL8A1, IFIT1, IFIT3 and p-EGFR in 50 NSCLC tissues by IHC.

	COL8A1		IFIT1		IFIT3	
	*r_s_*	*P* value	*r_s_*	*P* value	*r_s_*	*P* value
IFIT1	0.561	<0.001				
IFIT3	0.599	<0.001	0.660	<0.001		
p-EGFR	0.391	0.005	0.382	0.006	0.215	0.134

r, Spearman correlation.

## Discussion

Almost 25% of annual cancer incidence and cancer-related deaths worldwide are attributable to NSCLC (22), which is ranked as the leading cause of cancer-related deaths globally (23). Thus, early diagnosis and treatment of NSCLC is important, since clinical symptoms only become obvious at advanced stages of disease, and NSCLC exhibits relatively low sensitivity to targeted therapy, including gefitinib. Previous studies revealed that COL8A1 may function as an important player in several solid tumors (24, 25); however, whether COL8A1 is a pivotal player in NSCLC remains unclear. In the present study, we aimed to elucidate the biological functions of COL8A1 in NSCLC cells and explore the clinical significance and molecular mechanisms of COL8A1 in the occurrence, development, and metastasis of NSCLC. IHC assays were performed to evaluate COL8A1 expression levels in 94 NSCLC and 86 adjacent normal tissue samples and demonstrated that COL8A1 was aberrantly upregulated in NSCLC tissues, highlighting a potential role for COL8A1 as a novel prognostic biomarker of NSCLC, in which positive expression COL8A1 was associated with advanced pathological stage, consistent with a previous study (26).

To further evaluate the biological functions of COL8A1 *in vitro*, we measured the effects of its alteration on cell proliferation, invasion, cell cycle arrest, and apoptosis. The results confirmed that COL8A1 knockdown suppressed cell proliferation and invasion, and induced apoptosis and cell cycle arrest in A549 and H1975 cells. In contrast, overexpression of COL8A1 effectively contributed to cell growth and invasion, as well as inhibiting apoptosis in NSCLC cells. These data demonstrate that COL8A1 functions as an essential oncogene, regulating NSCLC cell progression *in vitro*. Additionally, COL8A1-silenced NSCLC cells exhibited significantly repressed growth *in vivo*.

As COL8A1 functions as an important oncogene in NSCLC development, we then clarified the underlying mechanisms by RNA sequence analysis, which identified multiple dysregulated genes involved in apoptosis, in addition to p53, interferon alpha response signaling and interferon gamma response signaling. We found ISGs genes were significantly regulated by COL8A1 according both in the RNA-seq and qPCR assay. It has been reported that IFIT1 and IFIT3 are critical for EGFR recycling and activation in oral squamous cell carcinoma (21). Interestingly, we found that COL8A1 could markedly enhance the phosphorylation of EGFR and AKT. So we next to confirm whether COL8A1 regulated the EGFR signaling pathway by IFIT1 and IFIT3. The ISGs, IFIT1 and IFIT3, are implicated in important functions in tumor development, and serve as biomarkers for various cancers (20). IFIT3 can promote IFN-α effector signaling, which in turn predicts IFN-α therapeutic response in patients with hepatocellular cancer (27). Based on these findings, we explored IFIT1 and IFIT3 functions in NSCLC development, and our results show that knockdown of IFIT1 or IFIT3 can inhibit NSCLC cell proliferation and invasion, promote apoptosis, indicating that these molecules act as oncogenes in NSCLC progression. Furthermore, our results show that knockdown of IFIT1 or IFIT3 can rescue the effects of COL8A1 on cell proliferation, migration, and apoptosis in NSCLC cell lines, indicating that COL8A1 contributes to NSCLC proliferation and invasion partly through regulating IFIT1 and IFIT3 expression, in turn, activation of EGFR. Regarding to the correlation analysis, we found that a weak correlation between COL8A1 and IFIT1, or IFIT3, might owe to the limited clinical sample size, which needed further explored to increase the sample size. Also, one limitation of this study is that the correlation between survival rate of lung cancer patients and the expression lf COL8A1, IFIT1/IFIT3 and phosphorylated EGFR were not analyzed, owing to the lack of the survival information of patients enrolled in this study, we will investigate this correlation in the future. Since IFIT1 and IFIT3 are functionally similar, clarification of the relationship between IFIT1 and IFIT3, based on their expression levels, warrants further investigation. Collectively, this study might provide a novel clue for targeted therapy of EGFR specific inhibitor in lung cancer patients.

In conclusion, our findings demonstrated that COL8A1 levels are clearly elevated in NSCLC tissues, as well as strongly associated with advanced stage of patients with NSCLC. *In vitro* studies revealed that COL8A1 influences NSCLC cell proliferation, invasion, and cell cycle, partly through regulation of IFIT1 and IFIT3 expression to affect EGFR activation, which function as ISGs, highlighting the novel roles of COL8A1 in NSCLC.

## Data Availability Statement

The original contributions presented in the study are included in the article/**Supplementary Material**. Further inquiries can be directed to the corresponding authors.

## Ethics Statement

The studies involving human participants were reviewed and approved by Ethics Committee of the Second Hospital of Lanzhou University. The patients/participants provided their written informed consent to participate in this study. The animal study was reviewed and approved by Institutional Animal Care and Use Committee of the Second Hospital of Lanzhou University.

## Author Contributions

YNZ and YXW initiated and designed this work. XZ and YPW conducted most of the experiments. LG and SW collected the tissue samples. YZ and XY performed the animal assay. LZ and LG performed apoptosis assay. JS and RL performed the IHC assay. SL and YYZ conducted data analysis. XZ and SW wrote the original manuscript. All authors contributed to manuscript revision, read, and approved the submitted version.

## Funding

This work was supported by the Natural Science Foundation, Gansu Province (17JR5RA221); Cuiying Science and Technology Innovation Project, the Second Hospital of Lanzhou University (CY2018-MS04), Gansu Province Higher Education Innovation Ability Improvement Project (2019B-011), and Lanzhou Science and Technology Development Guiding Plan Project (2019-ZD-63).

## Conflict of Interest

The authors declare that the research was conducted in the absence of any commercial or financial relationships that could be construed as a potential conflict of interest.

## Publisher’s Note

All claims expressed in this article are solely those of the authors and do not necessarily represent those of their affiliated organizations, or those of the publisher, the editors and the reviewers. Any product that may be evaluated in this article, or claim that may be made by its manufacturer, is not guaranteed or endorsed by the publisher.

## Supplementary Material

Supplementary Figure 1 Knockdown of COL8A1 suppresses NSCLC cell line progression. **(A)** Knockdown efficiency of lentivirus-mediated COL8A1-knockdown in H1299 cells determined by western blot assay. **(B)** Cell proliferation curves generated using cell viability assays in H1299 cells. **(C)** Representative image of the results of colony formation assays, showing crystal violet staining. **(D)** Cell cycle analysis examined by flow cytometry. **(E)** Cell apoptosis assays of H1299 cells. **(F)** Cell migration and invasion capacity of COL8A1-silenced NSCLC cells determined by transwell assays of H1299 cells. *p < 0.05, **p < 0.01, ***p < 0.001.

Supplementary Figure 2 knockdown of IFIT1/IFIT3 abolished the activation of EGFR medicated by COL8A1 overexpression. The expression of COL8A1, IFIT1, IFIT3, the phosphorylation of EGFR and the total EGFR were detected by western blotting assay.
