# Supplementary figures and images for: COL8A1 Promotes NSCLC Progression Through IFIT1/IFIT3-Mediated EGFR Activation
Source: Front Oncol. 2022 Feb 24;12:707525. doi: 10.3389/fonc.2022.707525 (PMC8907630; doi:10.3389/fonc.2022.707525)

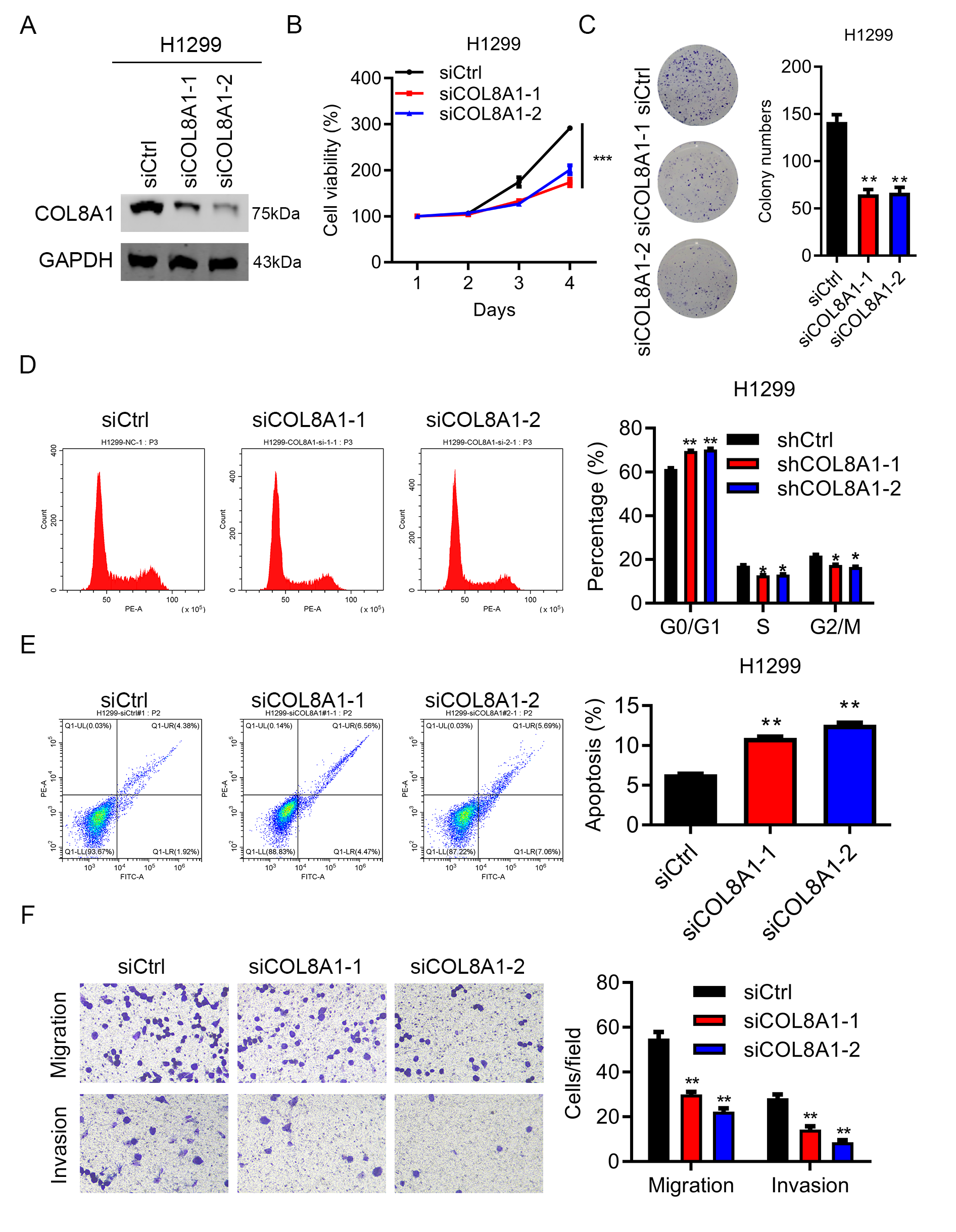

Supplement: Supplementary Figure 1 — Knockdown of COL8A1 suppresses NSCLC cell line progression. (A) Knockdown efficiency of lentivirus-mediated COL8A1-knockdown in H1299 cells determined by western blot assay. (B) Cell proliferation curves generated using cell viability assays in H1299 cells. (C) Representative image of the results of colony formation assays, showing crystal violet staining. (D) Cell cycle analysis examined by flow cytometry. (E) Cell apoptosis assays of H1299 cells. (F) Cell migration and invasion capacity of COL8A1-silenced NSCLC cells determined by transwell assays of H1299 cells. *p < 0.05, **p < 0.01, ***p < 0.001. [file Image_1.tif]

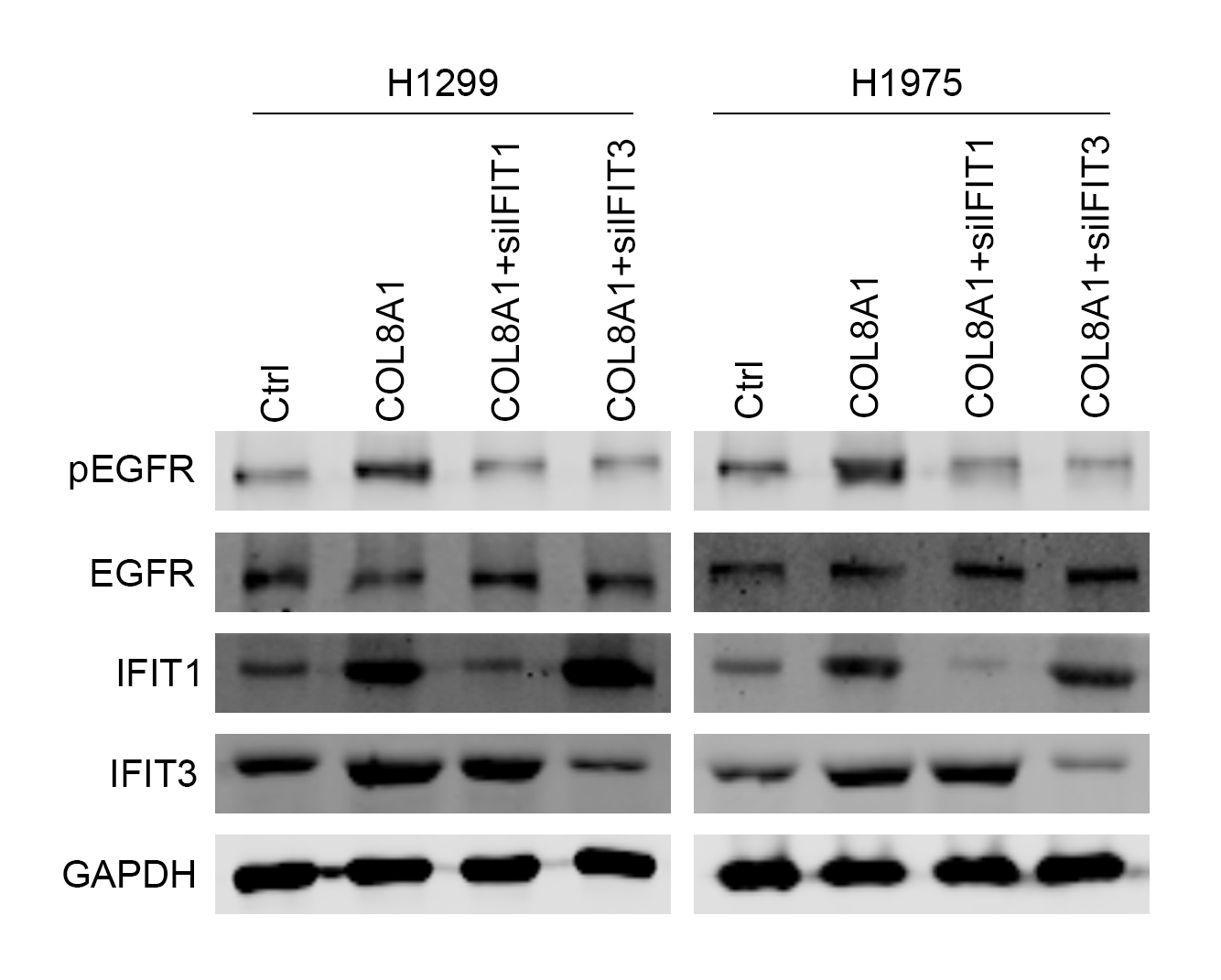

Supplement: Supplementary Figure 2 — knockdown of IFIT1/IFIT3 abolished the activation of EGFR medicated by COL8A1 overexpression. The expression of COL8A1, IFIT1, IFIT3, the phosphorylation of EGFR and the total EGFR were detected by western blotting assay. [file Image_2.tif]

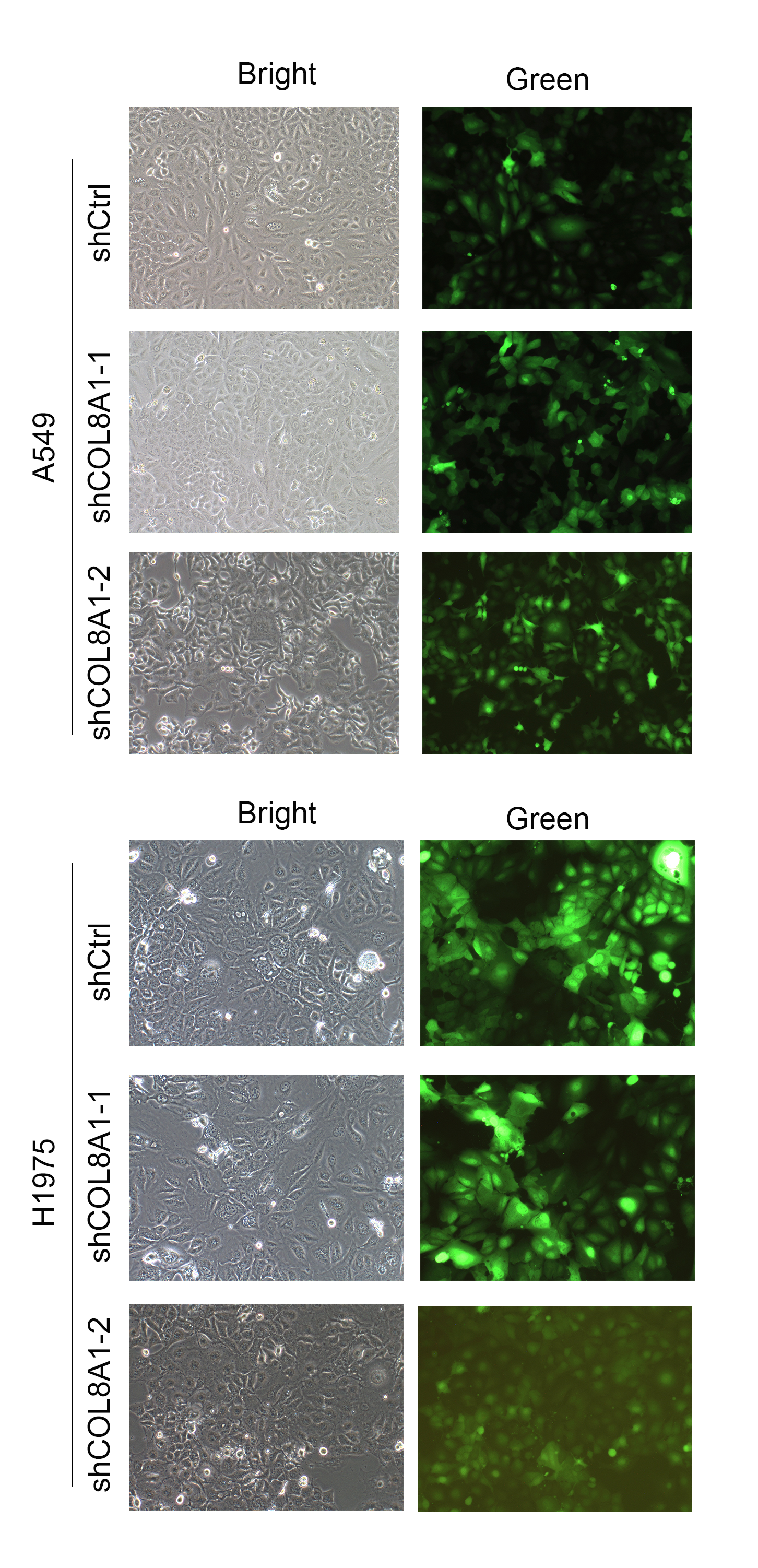

Supplement: Supplementary file 3 [file Image_3.tif]

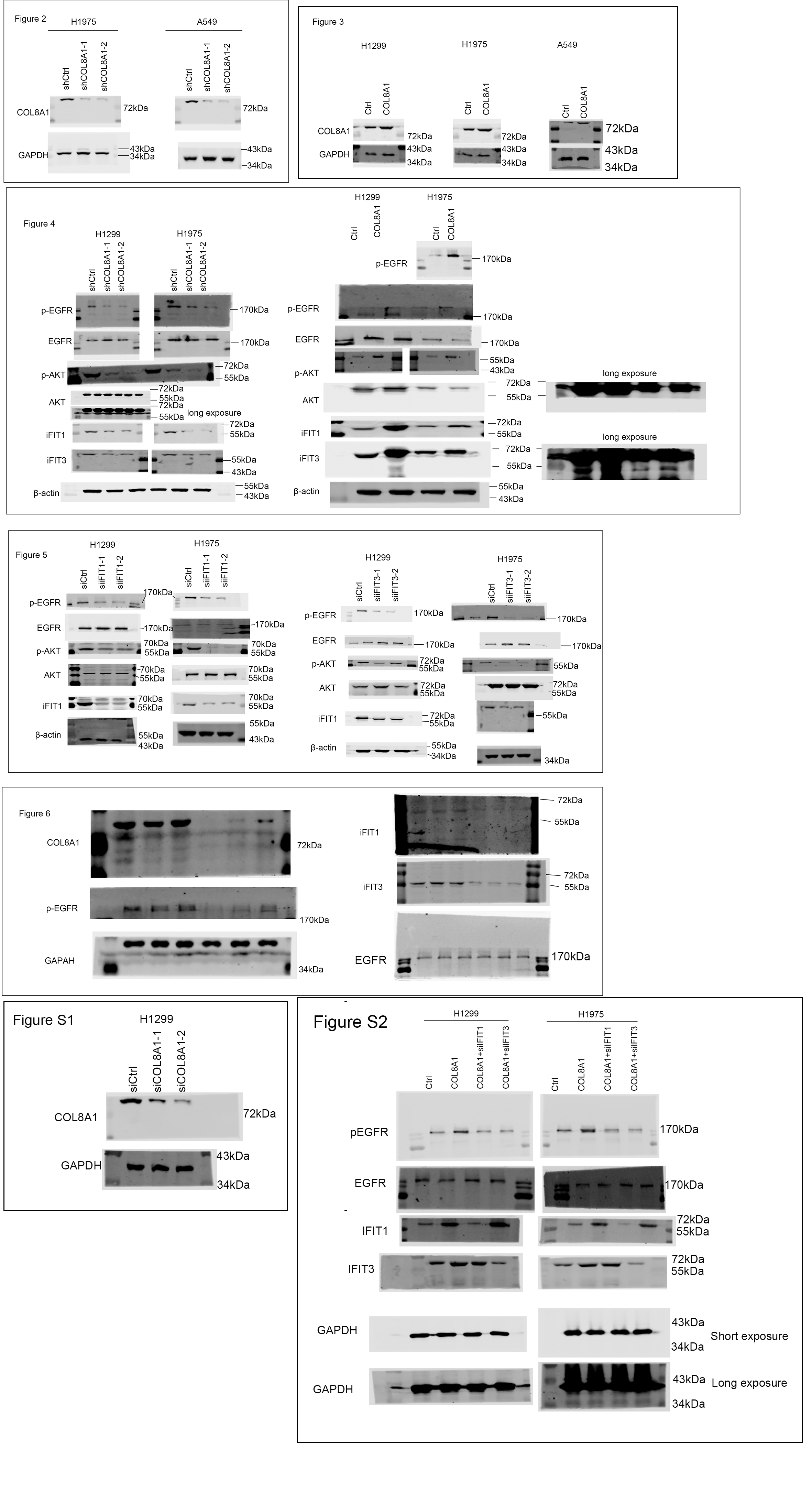

Supplement: Supplementary file 4 [file Image_4.tif]
