# Supplementary material for: COL8A1 Promotes NSCLC Progression Through IFIT1/IFIT3-Mediated EGFR Activation
Source: Front Oncol. 2022 Feb 24;12:707525. doi: 10.3389/fonc.2022.707525 (PMC8907630; doi:10.3389/fonc.2022.707525)
Supplement: Supplementary file 5 [file Table_1.docx]

**Supplementary Table 1**

**Real-Time PCR primer sequences**

|  | Forward (5’-3’) | Reverse (5’-3’) |
| --- | --- | --- |
| COL8A1 | AGAACTACAACCCGCAGAC | TTGAATAGAGCAACCCACA |
| IFIT1 | TAGCCAGATCTCAGAGGAGCC | CCATTTGTACTCATGGTTGCTGT |
| IFIT3 | GACAGGAAGACTTCTGAAGAACA | GACCTCACTCATGACTGCCC |
| IF44L | GACTTCTCAAAGCCGGGTCA | CCTTCATGGGGTCCAGTTCC |
| IFIT2 | CTGCAACCATGAGTGAGAACAA | CCTCCATCAAGTTCCAGGTGAA |
| IL4I1 | CACCAAGAGCTGGAGACACCAT | CTGCGTTCAGCCTTCCAGTC |
| IRAK3 | ATTACTTTGGTCCTGGGCAC | TCAACACTGCTCCATAGTTTGT |
| IFI35 | GATGACCCCAAAGTGGCTGA | CCACTCAACTGGCTGGACAT |
| IFI44 | GACTTCTCAAAGCCGGGTCA | CCTTCATGGGGTCCAGTTCC |
| IFIT5 | CGCTGCCATCATGAGTGAAAT | AGAAATTCAAGCTGTTGCCCA |
| IRF7 | GCTGTGCTGGCGAGAAGG | GGAGTCCAGCATGTGTGTGT |
| IFIH1 | AGATGCAACCAGAGAAGATCCA | TGGCCCATTGTTCATAGGGT |
| IL34 | TTAGGCACCTGCTTGGGGTC | CTCAGTGTGAAATCTGGCTCTGT |
| IL27RA | GGGAATTTCACTGTCGGGGT | AGGGGTGCTAATTCCTCCCT |
| TRIL | GGGCCTGGAGAGTCTAGTCA | TTCTTGCCCAGAAAGCGGAT |
| IL37 | TAGAAGACCCGGCTGGAAGC | TCACCTTTGGACTTGTGTGAAC |
| IIFITM3 | ACCGCCAAGTGCCTGAAC | TGAGCAGAATGGTCATGAGGAT |
| IL5 | AGCCAATGAGACTCTGAGGAT | TTTCCACAGTACCCCCTTGC |
| IRF5 | GAAAGCGAGCTCGGACCC | TCCCCGTTGACCCATTGAAG |
| IL23A | ACTAGTGGGACACATGGATCT | CTGGTGGATCCTTTGCAAGC |
| β-actin | GAGCTGCGTGTGGCTCCC | CCAGAGGCGTACAGGGATAGCA |
